# Polysaccharide from *Atractylodes macrocephala* Koidz binding with zinc oxide nanoparticles: Characterization, immunological effect and mechanism

**DOI:** 10.3389/fnut.2022.992502

**Published:** 2022-09-15

**Authors:** Ruonan Bo, Xiaopan Liu, Jing Wang, Simin Wei, Xinyue Wu, Ya Tao, Shuya Xu, Mingjiang Liu, Jingui Li, Huan Pang

**Affiliations:** ^1^College of Veterinary Medicine, Yangzhou University, Yangzhou, China; ^2^Jiangsu Co-innovation Center for Prevention and Control of Important Animal Infectious Diseases and Zoonoses, Yangzhou, China; ^3^Joint International Research Laboratory of Agriculture and Agri-Product Safety, The Ministry of Education of China, Yangzhou University, Yangzhou, China; ^4^School of Chemistry and Chemical Engineering, Yangzhou University, Yangzhou, China

**Keywords:** polysaccharide from *Atractylodes macrocephala* Koidz, zinc oxide nanoparticles, immunostimulatory activity, TLR4 signaling pathways, potential immunostimulator

## Abstract

*Atractylodes macrocephala* Koidz (*A. macrocephala*) has been used both as a traditional medicine and functional food for hundreds of years in Asia. And it has a variety of biological activities, such as enhancing the ability of immunity and modulating effect on gastrointestinal motility. In this study, a water-soluble polysaccharide with molecular weight of 2.743 × 10^3^ Da was isolated from the root of *A. macrocephala*. Polysaccharide from *A. macrocephala* (AMP) consisted of arabinose, galactose, glucose, xylose, mannose, ribose, galactose uronic acid, glucose uronic acid, with a percentage ratio of 21.86, 12.28, 34.19, 0.43, 0.92, 0.85, 28.79, and 0.67%, respectively. Zinc plays an important role in immune system. Therefore, we supposed that AMP binding with zinc oxide (ZnO) nanoparticles (AMP-ZnONPs) might be an effective immunostimulator. AMP-ZnONPs was prepared by Borch reduction, and its structural features were characterized by Scanning Electron Microscope (SEM), Transmission electron microscope (TEM), TEM-energy dispersive spectroscopy mapping (TEM-EDS mapping), Fourier transform infrared spectroscopy (FT-IR), X-ray photoelectron spectrometer (XPS), X-ray diffraction (XRD), particle size and zeta-potential distribution analysis. Then, its immunostimulatory activity and the underlying mechanism were evaluated using RAW264.7 cells. The results showed that AMP-ZnONPs remarkably promoted cell proliferation, enhanced phagocytosis, the release of nitric oxide (NO), cytokines (IL-6 and IL-1β) and the expression of co-stimulatory molecules (CD80, CD86 and MHCII). Moreover, AMP-ZnONPs could promote the expression of Toll-like receptor 4 (TLR4), Myeloid differentiation factor 88 (MyD88), TNF receptor associated factor 6 (TRAF6), phospho-IκBα (P-IκBα) and phospho-p65 (P-p65), and TLR4 inhibitor (TAK242) inhibited the expression of these proteins induced by AMP-ZnONPs. Therefore, AMP-ZnONPs activated macrophages by TLR4/MyD88/NF-κB signaling pathway, indicating that AMP-ZnONPs could act as a potential immunostimulator in medicine and functional food.

## Introduction

In recent years, immunoregulatory polysaccharides are considered important macromolecules for stimulation of immune response, then gradually become a major research hot spot (1, 2). *Atractylodes macrocephala* Koidz *(A. macrocephala)* has been used both as traditional medicine and functional food for hundreds of years in Asia, and it was approved as a functional food by the National Health Commission of the People’s Republic Health of China (3–5). Polysaccharide from *A. macrocephala* (AMP) has a variety of biological activities, such as enhancing the ability of immunity, modulating effect on gastrointestinal motility and decreasing the blood glucose level (6–8). However, a lot of natural polysaccharides exhibit only weak bioactivities due to the limitation of structural and conformational properties (9). Thus, further research about enhancing bioavailability of AMP is necessary.

Zinc (Zn) deficiencies in the body is a serious problem, which severely harms the health of the organism and causes the etiology of myocardial apoptosis, deregulated homeostasis (10–12). In addition, zinc is important for cellular homeostasis and also serves as a regulatory signaling molecule for immune cells (13, 14). Zinc oxide (ZnO) is listed as “commonly considered as safe” by the US Food and Drug Administration (FDA) (15). Some studies have shown that Zn has a significant role in the development and activation of effector cells of the innate and adaptive immune systems (16–18). ZnO nanoparticles (ZnONPs) have been exploited in biomedical and preclinical research for their advantages such as non-toxicity and low cost (19, 20). However, ZnONPs are limited their application in drug delivery due to their poor water solubility, strong agglomeration and less dispersion. Hence, it is imperative to develop an effective, safe and high-content Zn-supplement. To improve the dispersion of particles in water, a silane coupling agent (KH550) was used to modify the ZnONPs (21). In addition, KH550 was easily grafted at the ZnONPs interface, and the other side of KH550 carries an amino group that was easily grafted with polysaccharides.

Macrophages are one of the most important effector cells of the immune system, and play pivotal roles in the immune response (22). Phagocytosis is a marker of their activation. Upon activation, macrophages release NO, diverse cytokines and co-stimulatory molecules (23, 24). Many natural polysaccharides modulate the immune system through the activation of TLR4 in macrophages (25–29). Toll-like receptor 4 (TLR4), a key pattern recognition receptor involved in the activation of macrophages, is reported the major component of the signaling including nuclear factor-κB (NF-κB) signaling pathway.

**FIGURE 1:**
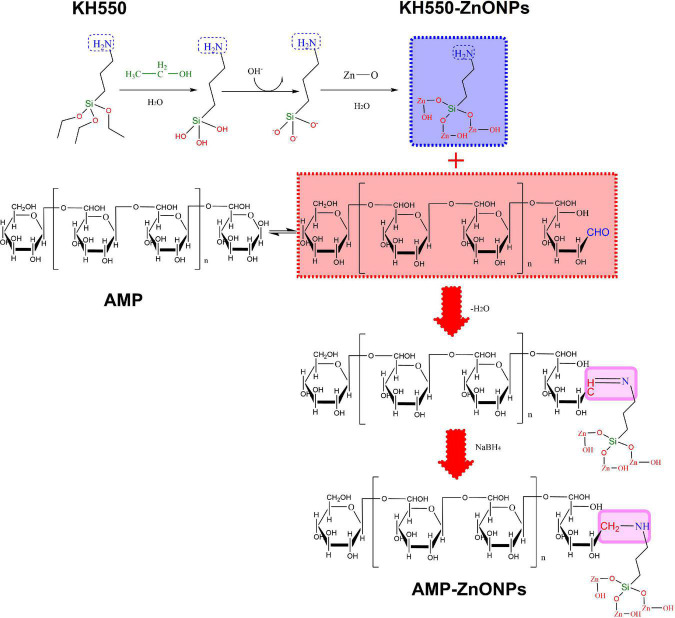
Schematic illustration of AMP-ZnONPs synthesis. The surface of ZnONPs was modified with KH550 through Borch reduction and then AMP was bond to the KH550-ZnONPs.

We supposed that AMP binding with ZnONPs (AMP-ZnONPs) might provide a novel way to explore an effective immunostimulator. To enhance the dispersive capacity of ZnONPs in the water, *γ*-aminopropyltriethoxy silane (KH550) was applied to modify its surface. Then, AMP-ZnONPs was successfully prepared by the binding of KH550-ZnONPs and AMP *via* the Borch reduction between -NH_2_ group and -CHO group (Figure 1). Its structural properties were characterized and then its immunostimulatory activities including cell viability, phagocytosis, surface molecules, cytokines release were evaluated using RAW264.7 cells. To further reveal the mechanism of immune stimulation, the effects of AMP-ZnONPs on the TLR4/MyD88/NF-κB signaling pathways were analyzed. This study is expected to provide new ideas for the development and utilization of polysaccharides and microelements in the food and pharmaceutical industry.

## Materials and methods

### Reagents and materials

*A. macrocephala* was purchased from the Tongrentang Company in Beijing. The plant material was identified by Prof. Jingui Li. The purified AMP was prepared in our laboratory and the polysaccharide content was 96% (UV). RAW264.7 cells were obtained from American Type Culture Collection (ATCC, Rockville, MD, United States). Fucose (Cat No. B25632), arabinose (Cat No. B65342), rhamnose (Cat No. B50770), galactose (Cat No. B21893), glucose (Cat No. B21882), xylose (Cat No. B21880), mannose (Cat No. B21895), fructose (Cat No. B21896), ribose (Cat No. B21897), galacturonic acid (Cat No. B21894) and glucuronic acid (Cat No. B25302) were purchased from Shanghai Ye Yuan Biotechnology Co., Ltd. FITC-dextran (Cat No. 60842-46-8) and fetal bovine serum (Cat No. F8318) were purchased from Sigma Corporation of America. DMEM culture solution (Cat No. SH30022.01) was purchased from HyClone. NO test kit (Cat No. S0021) was obtained from Beyotime Biotechnology. Silane coupling agent (KH550, Cat No. A7440) and ZnONPs (purity 99.9%, Cas No. 1314-13-2) were the products of Sinopharm Chemical Reagent Ltd. RNA-easy Isolation Reagent (Cat No. R701) was purchased from Vazyme Biotech Co., Ltd. The CCK8 (Cat No. 40203ES80), Hifair^®^ III 1st Strand cDNA Synthesis SuperMix for qPCR (gDNA digester plus) (Cat No. 11141ES60) and Hieff^®^ qPCR SYBR Green Master Mix (High Rox Plus) (Cat No. 11184ES08) were the products of Yeasen Biotech Co., Ltd. Rabbit Anti-CD80 Polyclonal Antibody (Cat No. bs-1479R), Rabbit Anti-CD86 Polyclonal Antibody (Cat No. bs-1035R) and Rabbit Anti-MHC Class II/HLA DMB Polyclonal Antibody (Cat No. bs-4107R) were purchased from Biosynthesis Biotechnology Inc. (Beijing, China). Antibodies of TLR4 (Cat No. 14358s), TRAF6 (Cat No. 67591s), MyD88 (Cat No. 4283s), phospho-IκBα (P-IκBα) (Cat No. 4812s), phospho-p65 (P-p65) (Cat No. 8242s) and β-actin (Cat No. 4970s) were the products of Cell Signaling Technology Pathways. TLR4 inhibitor (TAK242, Cat No. M4838) was purchased from Abmole (Houston, TX, United States).

### Extraction and purification of polysaccharide from *Atractylodes macrocephala*

AMP was extracted by water extraction and alcohol precipitation methods. Briefly, *A. macrocephala* was first extracted with alcohol for 2 times to remove the impurity. Second, *A. macrocephala* was decocted in water. The aqueous extract was concentrated under a vacuum. After that, a threefold volume of alcohol was added, the precipitated was washed three times with anhydrous ethanol, acetone and diethyl (30). Then, the protein was removed using sevage methods (31). The polysaccharide was dialyzed for 24 h. We further purified the crude polysaccharide through Sephadex G-100 column. Finally, the purified extraction was lyophilized and the polysaccharide content of AMP was determined by UV-VIS absorption spectrometry.

### Molecular weight measurement of polysaccharide from *Atractylodes macrocephala*

The molecular weight of purified samples was determined by high-performance gel permeation chromatography (HPGPC; Agilent 1,260 Infinity). Three gel permeation columns (KS-805, KS-804 and KS-802) were linked in serials. The column temperature was kept at 70°C. Double distilled water was used as mobile phase and the flow rate was kept at 1 mL⋅min^–1^. The calibration curve was constructed using different molecular weights of Dextran standards, and the molecular weight of AMP was calculated by Dextran standards.

### Monosaccharide composition analysis of polysaccharide from *Atractylodes macrocephala*

5 mg of AMP and 1 mL of trifluoroacetic acid (TFA, 2 M) were hydrolyzed at 121°C for 2 h. The mixture was dried with nitrogen, and then washed with methanol 2–3 times followed. The monosaccharide standards included fucose, arabinose, rhamnose, galactose, glucose, xylose, mannose, fructose, ribose, galacturonic acid and glucuronic acid. Finally, samples were analyzed *via* high-performance anion-exchange chromatography (HPAEC) (ICS5000, Thermo Fisher Scientific, United States) with Dionex™ CarboPac™ PA-20 column (150 mm × 3.0 mm, 10 μm). Mobile phase A was 0.1 M NaOH, mobile phase B was 0.1 M NaOH, 0.2 M NaAc. The composition of eluent A was adjusted to 95% at 0 min, 80% at 30 min, 60% at 30.1 min, 60% at 45 min, 95% at 45.1 min, 95% at 60 min. The column temperature was 30°C. The flow rate was 0.5 mL⋅min^–1^ and the injection volume was 5 μL. The determination of monosaccharide composition was made with an electrochemical detector and the peaks were processed using Chromeleon 7.2 CDS (Thermo Scientific).

### Preparation of AMP-ZnONPs

In order to fully hydrolyze KH550, 4 mL KH550 was added to 400 mL of equal volumes of alcohol and water, the mixture was reacted for 10 min under ultrasonication, and then agitated for 20 min on a magnetic stirrer. The pH of the solution was adjusted to between 6.5 and 7.0 with 0.2 M HCl to generate silicon-oxygen bonds for grafting the ZnONPs. Then, 4.5 g of ZnONPs was added to this solution, sonicated for 30 min, agitated for 30 min at 200 rpm on a magnetic stirrer (80°C), and then the mixture was collected and lyophilized. The surface of ZnONPs was modified with KH550 by these processes. KH550-ZnONPs (10 mg) was added to water (20 mL). After sonicating for 1 h, AMP was added to the KH550-ZnONPs (m_AMP_: m_KH550–ZnONPs_ = 4:1) and stirred for 24 h. The -CHO of AMP and the -NH_2_ of ZnONPs were linked to assemble AMP-ZnONPs by Borch reduction.

### Characterization of AMP-ZnONPs

Scanning Electron Microscope (SEM, Zeiss Supra55, Germany) was used to detect the samples of ZnONPs, KH550-ZnONPs, AMP and AMP-ZnONPs, the image magnification was 5,000 x. The morphology of samples was also observed *via* a Transmission electron microscope (TEM, HT7800, Hitachi, Japan). The element distribution was observed by Transmission electron microscope-energy dispersive spectroscopy mapping (TEM-EDS mapping, Tecnai G2 F30 S-TWIN, FEI, US) to verify the connection of the ZnONPs and AMP. The Fourier transform infrared spectroscopy (FT-IR, Thermo Electron Corporation, United States) spectra were recorded in the mid-infrared region. The samples were determined at room temperature on an X-ray diffraction (XRD, D8 Advance, Germany), and operated at 40 kV and 40 mA. The samples were determined with X-ray photoelectron spectrometer (XPS, ESCALAB 250Xi, United States). Data were analyzed using the Avantage software. The laser particle size analyzer (NanoPlus 3, Micromeritics Instrument Corp., United States) was applied to measure average particle size, polydispersity index (PDI) and zeta-potential.

### Cell culture

RAW264.7 cells were cultured in the Dulbecco’s modified Eagle’s medium (DMEM) with 10% fetal bovine serum. Cells were maintained under a humified atmosphere at 37°C with 5% CO_2_.

### Cell activity assay

The cell activity of AMP and AMP-ZnONPs on RAW264.7 cells was determined according to the CCK-8 method. Cell viability of RAW264.7 cells was evaluated after treatment with AMP and AMP-ZnONPs (0.06–250 μg⋅mL^–1^) for 24 h. Following this, the supernatants were discarded, then added fresh DMEM medium (100 μL⋅well^–1^) containing CCK8 (10 μL⋅well^–1^) and cultivated for 1.5–4 h at 37°C. Finally, the absorbance at 450 nm was measured by microplate reader. The cell survival rate was calculated as follows:


$$\text{Cell activity }\left(\%\right)={\text{A}}_{\text{2}}/{\text{A}}_{\text{1}}\times \text{1}00\%$$

(Where A_1_ and A_2_ are the absorbances of the control and test samples, respectively).

### Measurement of nitric oxide

In brief, RAW264.7 cells (1 × 10^5^ cells⋅mL^–1^) were separately exposed to ZnONPs, AMP-ZnONPs and AMP (1.95 μg⋅mL^–1^) for 24 h at 37°C in a constant temperature incubator ventilating with 5% CO_2_. At the end of incubation, the NO amount in the supernatant was measured by a Griess reagent system kit K.

### Quantitative real-time polymerase chain reaction

Real-time polymerase chain reaction (PCR) was employed for the determination of cytokines (IL-6 and IL-1β) and TLR4, MyD88, TRAF6 mRNA expression. The total RNA of RAW264.7 cells was obtained with Trizol reagent, and then synthesized the corresponding cDNA. The Hieff qPCR SYBR Green Master Mix was employed to perform Quantitative real-time PCR assay. The primer sequences of genes were displayed in Table 1. The following PCR protocol was referenced by our previous report (32). The expression of each gene was analyzed by the 2^–ΔΔct^ comparative method.

**TABLE 1:** The primer sequences of target genes.

Gene	Sense (5′–3′)	Antisense (5′-3′)
IL-6	TTCCATCCAGTTGCCTTCTTG	AATTAAGCCTCCGACTTGTGAA
IL-1β	ATCTCGCAGCAGCACATCA	CCAGCAGGTTATCATCATCATCC
TLR4	TGGTCAGTGTGATTGTGGTATC	GCTTTCTCCTCTGCTGTACTT
MyD88	TCGATGCCTTTATCTGCTACTG	GGTCGGACACACACAACTTA
TRAF6	GCTGAGCCACAATACTCACTAA	TTCTAGCGGATGGACATTACAC
GADPH	ATGGTGAAGGTCGGTGTGAA	CCTTGACTGTGCCGTTGAAT

### Determination of phagocytic function using CytExpert flow cytometer

RAW264.7 cells (1 × 10^6^⋅mL^–1^) were cultured in 6 well plates at 37°C for 12 h, and then exposed to ZnONPs, AMP, AMP-ZnONPs (1.95 μg⋅mL^–1^) or LPS (0.5 μg⋅mL^–1^) for 24 h, respectively. The cells were incubated with 1 mg⋅mL^–1^ FITC-dextran for 1 h, then the reaction was stopped by cold PBS. The FITC-dextran intensity of cell samples was analyzed by CytExpert flow cytometer (Beckman Coulter, CA, United States).

### High-resolution laser confocal microscopy

Cells were seeded at 1 × 10^5^ cells⋅mL^–1^ on coverslips in a 24 well plate, then stimulated with ZnONPs, AMP, AMP-ZnONPs (1.95 μg⋅mL^–1^) or LPS (0.5 μg⋅mL^–1^), and incubated with 1 mg⋅mL^–1^ FITC-dextran (1 mg⋅mL^–1^) at 37°C for 1 h. After incubation, RAW264.7 cells were fixed with 4% paraformaldehyde for 10 min. Cell samples were stained with phalloidin for 1 h under dim light, followed by DAPI staining. The green fluorescence was measured by laser confocal microscopy (LSCM) (TCS SP8 STED, Germany).

### Expression of cell surface molecule CD80, CD86, and MHCII

The cells (1 × 10^6^ cells⋅mL^–1^) were treated with AMP-ZnONPs for 24 h in a 6-well plate. Then, the cells were suspended and incubated with anti-CD80, anti-CD86 and anti-MHCII at 4°C for 30 min, and analyzed by the CytExpert flow cytometer.

### Cell morphological observation

The cells (1 × 10^5^ cells⋅mL^–1^) were plated on coverslips in 24-well plates, and then treated with ZnONPs, AMP, AMP-ZnONPs (1.95 μg⋅mL^–1^), LPS (0.5 μg⋅mL^–1^) or DMEM. The RAW264.7 cells with glutaraldehyde-treated were prepared for 24 h. Next, the cells were evaporated using a 30, 50, 70, 80, 90, 95, and 100% ethanol gradient (10–15 min each time); then displaced in Na_2_SO_4_, dried at a tipping point, and finally scanned by SEM (HT7800, Hitachi, Japan) at 1,000 and 8,000×.

### Western blotting analysis

The BCA protein assay kit was employed to detect the protein concentrations. Equal amounts (30 μg) of total protein were separated and transferred to the NC membrane (33). The membrane was incubated with 5% skim milk for 2 h and then incubated with gentle shaking with primary antibodies at 4°C overnight. Later incubated NC at 4°C with antibodies of TLR4 (rabbit, 1: 1,000), TRAF6 (rabbit, 1: 1,000), MyD88 (rabbit, 1: 1,000), P-p65 (rabbit, 1: 1,000), P-IκBα (rabbit, 1: 1,000) and ß-actin (rabbit, 1: 1,000). After incubation with the primary antibody, the membrane was exposed to goat anti-rabbit secondary antibody (1: 5,000) at room temperature for 1 h. The membrane was washed with TBST for 3 times, membrane-bound antibodies were visualized using the ECL Enhanced Chemiluminescence system, the protein band intensity was analyzed with Image J Analysis Software.

### Statistical analysis

GraphPad Prism 5.0 Software was utilized for statistical analysis. Data were analyzed by one-way analysis of variance (ANOVA) followed by Dunnett’s multiple comparisons test. All the data were expressed using mean ± standard deviation (SD). The criterion of significance was *P* < 0.05.

## Results and discussion

### Molecular weight and monosaccharide composition of polysaccharide from *Atractylodes macrocephala*

**FIGURE 2:**
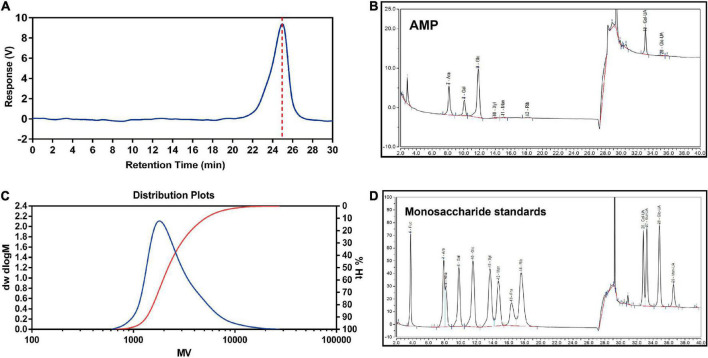
Molecular weight and monosaccharide composition of AMP. The peak at retention time of AMP **(A)** and molecular weight distribution **(B)** of AMP. HPAEC chromatograms of AMP **(C)** and monosaccharide standards **(D)**. Fuc, fucose; Ara, arabinose; Rha, rhamnose; Gal, galactose; Glc, glucose; Xyl, xylose; Man, mannose; Fru, fructose; Rib, ribose; Gal-UA, galacturonic acid; Glu-UA, glucuronic acid.

As shown in Figure 2A, the results of HPGPC implied a good homogeneity of AMP. The retention time was 24.98 min. The chromatographic result of HPGPC showed that AMP had one peak, which indicated that AMP was homogeneous polysaccharides. Based on the regression equation of the dextran standard curve, y = 11.864079–0.338831×–0.005080×^2 + 0.000195×^3, the molecule weight of AMP was calculated as 2.743 × 10^3^ Da (Figure 2B). The result of monosaccharide composition obtained from AMP was described by HPAEC (Figures 2C,D). AMP was composed of arabinose, galactose, glucose, xylose, mannose, ribose, galactose uronic acid, glucose uronic acid, with a percentage ratio of 21.86, 12.28, 34.19, 0.43, 0.92, 0.85, 28.79, and 0.67%, respectively.

### Characteristics of AMP-ZnONPs

#### Morphological characteristics of AMP-ZnONPs

**FIGURE 3:**
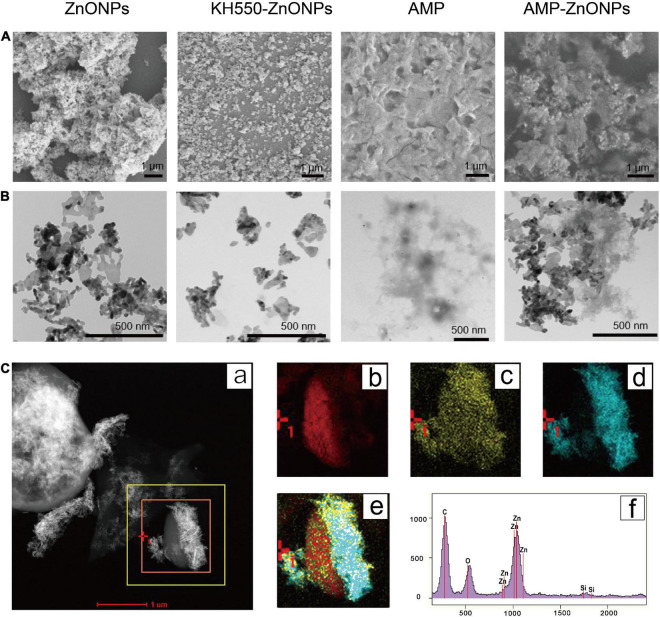
The SEM analyzes of ZnONPs, KH550-ZnONPs, AMP and AMP-ZnONPs **(A)**. The TEM analyzes of ZnONPs, KH550-ZnONPs, AMP and AMP-ZnONPs **(B)**. TEM-EDS mapping image of AMP-ZnONPs **(C)**, TEM image (a), corresponding elements distribution of C (b), O (c) and Zn (d), merge (e), EDS spectrum (f).

Untrastructure of ZnONPs, KH550-ZnONPs, AMP and AMP-ZnONPs was obtained with the SEM (Figure 3A). The AMP exhibited an irregular surface with many folds. ZnONPs showed rod morphology and a nano-lamellar structure. The KH550-ZnONPs after the surface modification displayed particles with uniform size, good monodispersity, and no obvious agglomeration. The surface of AMP appeared to be covered by rod-shaped ZnONPs, which was attributed to the strong interaction between amino in ZnONPs and hemiacetal in AMP because of hydroamination. Ultrastructure of ZnONPs, KH550-ZnONPs, AMP and AMP-ZnONPs as shown in Figure 3B. The ZnONPs showed strong clustering and exhibited aggregation. The KH550-ZnONPs had smaller clusters and showed a homogenous dispersion, all aggregation was disrupted. As shown in the AMP-ZnONPs, KH550-ZnONPs were connected to the surface of AMP. The TEM-EDS mapping and EDS spectra showed that C, O and Zn elements were present in AMP-ZnONPs (Figure 3C). Therefore, the results strongly supported the formation of AMP-ZnONPs.

#### Fourier transform infrared spectroscopy analysis

**FIGURE 4:**
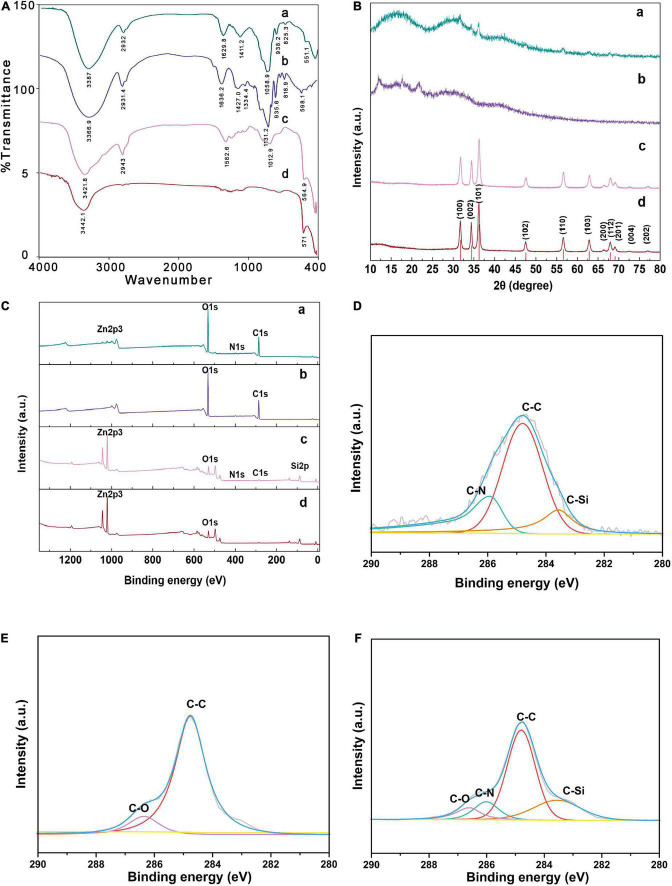
Spectroscopic characterization and curve-fitting spectra of AMP-ZnONPs. The FT-IR pattern **(A)**, XRD pattern **(B)**, XPS spectra **(C)** of AMP-ZnONPs (a), AMP (b), KH550-ZnONPs (c) and ZnONPs (d). The curve-fitting of C1s **(D)** spectra of KH550-ZnONPs. The curve-fitting of C1s **(E)** spectra of AMP. The curve-fitting of C1s **(F)** spectra of AMP-ZnONPs.

As shown in Figure 4A, FT-IR spectra of AMP revealed that the characteristic peaks of polysaccharide at 3366.9 cm^–1^, 2931.4 cm^–1^, and 1427 cm^–1^ were attributed to O-H stretch vibration of hydroxyl group, C-H stretch vibration and O-H bending vibration, respectively (34). Moreover, the weak bands around 935.6 cm^–1^ and 818.9 cm^–1^ indicated that there were α-configuration and β-configuration (35, 36). FT-IR spectra of ZnONPs showed an intense peak at 571.1 cm^–1^ and a broad peak at 3442.1 cm^–1^. The weak absorption peak at 3442.1 cm^–1^ could be attributed to stretching vibration of associating hydroxyls formed by weak hydrogen bonding as well as van der Waals interaction (37). The stretching vibration band at 571.1 cm^–1^ corresponding to the Zn-O bond (38). Compared with ZnONPs, KH550-ZnONPs displayed a new typical characteristic absorption peak of -NH_2_ at 1582.6 cm^–1^, which was attributed to the characteristic absorption peak of KH550. The AMP-ZnONPs spectra showed the characteristic absorption peaks of ZnONPs, KH550 and AMP. In addition, the signal in 1058.9 cm^–1^ was mainly assigned to the stretching vibration of the C-O-C group, the absorption peak at 1,629 cm^–1^ corresponded to N-H stretching vibrations (39, 40). FT-IR spectra of AMP showed that the band at 1636.2 cm^–1^ corresponding to the C = O bond (41). There was no N-H bond in AMP, after connecting to ZnONPs, the presence of an N-H bond in AMP-ZnONPs indicated a possible connection. The result provided evidence for the successful grafting of the KH550-ZnONPs by the NH_2_ groupings in KH550 agents onto AMP.

#### X-ray diffraction analysis

XRD, as a valuable instrument (42), could be used to further confirm the composition of AMP-ZnONPs (Figure 4B). The characteristic peaks located at 2θ = 31.7, 34.4, 36.2, 47.5, 56.6, 62.8, 66.3, 67.9, 69.1, 72.5, 76.9, corresponding to the (100), (002), (101), (102), (110), (103), (200), (112), (201), (004), (202) planes, respectively, match well with characteristic reflections of ZnONPs (P63mc, JCPDS no. 89-0511). A similar curve of ZnONPs modified by KH550 proved that modification of KH550 did no effect on the phase formation of ZnONPs. The XRD results of AMP recorded between 10° and 30° suggested the presence of crystalline components, with major reflections at 12.0°, 17.7° and 21.8°. This profile was also observed in other different polysaccharides (43, 44). The XRD profile of AMP-ZnONPs showed that the main characteristic peaks of ZnONPs, confirming that the hexagonal structure of the ZnONPs was not affected after binding with AMP. Moreover, as sreov-shapederot affected aft θ = 10–20, the modified AMP molecule maybe undergo a chemical structure change and convert to amorphous materials under this circumstance.

#### X-ray photoelectron spectrometer analysis

XPS was depicted in Figure 4C to investigate the surface compositions of the ZnONPs (45, 46). Compared with ZnONPs, the XPS spectra of the KH550-ZnONPs exhibited the characteristic peak components of Zn2p3, O1s, N1s, C1s and Si2p, suggesting that silane had successfully modified on the surface of ZnONPs. The AMP showed a Zn-free surface, the two peaks at 285, and 532 eV correspond to C1s and O1s, respectively. The C1s core-level spectrum of the KH550-ZnONPs was divided into three peak components: C-C, C-N and C-Si (Figure 4D). The C1s core-level spectrum of the AMP was divided into two peak components: C-C and C-O (Figure 4E). The peaks of C-O (286.6 eV), C-C (284.8 eV), C-N (286.3 eV) and C-Si (283.5 eV) were also observed in the C1s spectrum of the AMP-ZnONPs (Figure 4F), the C-N single bond peak was introduced by KH550, and the C-O peak was introduced by AMP. The presence of a C-N bond confirmed that there was a cross-linking reaction between the AMP and the KH550-ZnONPs. Although the Zn2p3 peak was reduced due to the AMP shielding, some active sites of ZnONPs remain even. These results were in good agreement with previous results of XRD and FT-IR, indicating that the successful grafting of AMP and KH550-ZnONPs.

#### Particle size, polydispersity index and zeta potential

**FIGURE 5:**
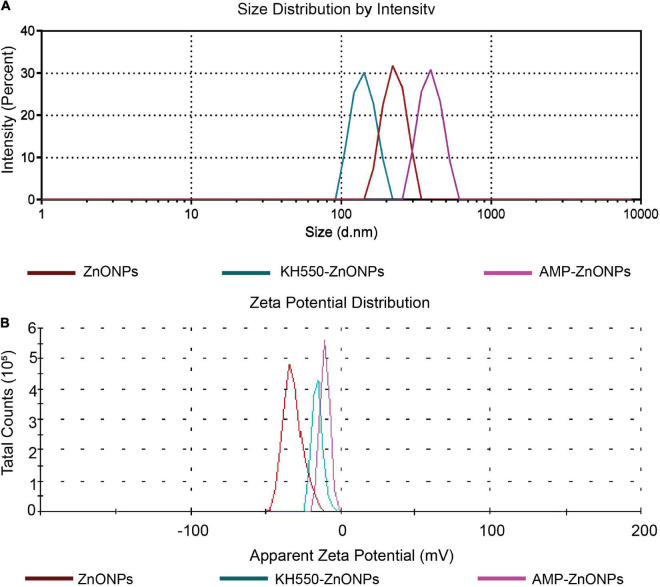
The particle size **(A)** and Zeta potential **(B)** of ZnONPs, KH550-ZnONPs and AMP-ZnONPs.

The particle size (Table 2 and Figure 5A) of the ZnONPs was larger than that of the KH550-ZnONPs, this result revealed that after KH550 being modified on the surface of ZnONPs promoted the particle size to be smaller. The particle size of AMP-ZnONPs was slightly larger than KH550-ZnONPs, which may be caused by the AMP binding on KH550-ZnONPs. The PDI values of AMP-ZnONPs was lower than 0.3, which was considered optimal for the dispersion and homogeneity (47). The zeta potential of AMP-ZnONPs was more negatively charged than KH550-ZnONPs (Figure 5B), with AMP-ZnONPs gaining additional negative charge of –4.43 mV. Negatively charged nanoparticles are more likely to be internalized by cells than positively charged nanoparticles, this property underlies the fact that AMP-ZnONPs stimulates phagocytosis of RAW264.7 cells more significantly than either ZnONPs or AMP alone.

**TABLE 2:** The particle size, PDI, and zeta potential of ZnONPs, KH550-ZnONPs and AMP-ZnONPs (*n* = 3).

Samples	ZnONPs	KH550-ZnONPs	AMP-ZnONPs
Size (nm)	211.89 ± 8.98	136.73 ± 3.12	391.37 ± 3.27
PDI	0.095 ± 0.053	0.391 ± 0.137	0.206 ± 0.086
Zeta potential (mV)	–33.20 ± 1.11	–10.97 ± 0.32	–15.40 ± 0.35

#### Cell viability

**FIGURE 6:**
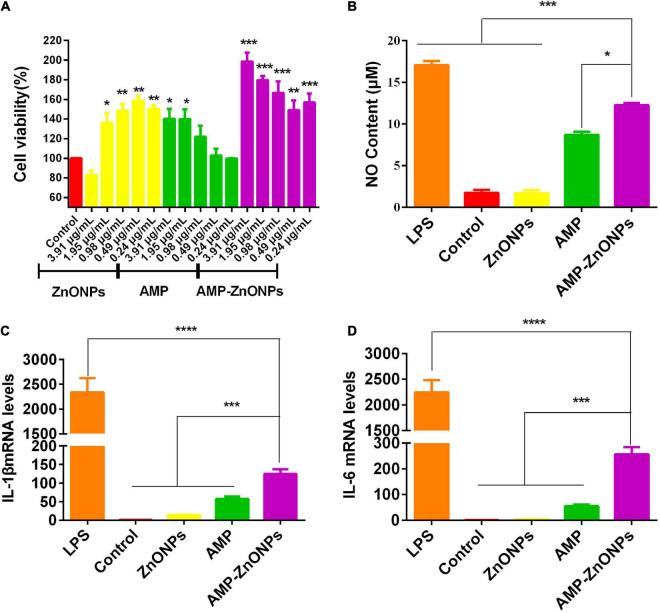
The proliferative activity of RAW264.7 cells stimulated by ZnONPs, AMP and AMP-ZnONPs **(A)**. NO content of cell culture supernatant **(B)** and the mRNA expression of IL-1β **(C)**, IL-6 **(D)** in RAW264.7 cells of different groups. (*P < 0.05, **P < 0.01, ***P < 0.001, ****P < 0.0001 vs. AMP-ZnONPs).

The cell viability of ZnONPs, AMP and AMP-ZnONPs on RAW264.7 cells were shown in Figure 6A. Compared with the control group, ZnONPs, AMP and AMP-ZnONPs exerted no damaging effect and promoted cell proliferation to a certain extent at a concentration of 0.24–1.95 μg⋅mL^–1^. When the concentration of AMP-ZnONPs was 0.49–3.91 μg⋅mL^–1^, the proliferation effect was proportional to the concentration. To compare the immune effects of ZnONPs, AMP and AMP-ZnONPs at the same concentration level, the concentration of ZnONPs, AMP and AMP-ZnONPs at 1.95 μg⋅mL^–1^ were chosen in the following experiments.

#### AMP-ZnONPs induced cells nitric oxide production

NO is an important active substance associated with the immunomodulatory effect (48), which participates in apoptosis regulation and host defense function (49). The NO production was calculated from the standard curve formula. The results of NO release in AMP-ZnONPs were shown in Figure 6B. As a positive control, the NO content of LPS group (0.5 μgPS ^–1^) showed significantly higher than the control group (*P* < 0.001). And the release of NO in AMP-ZnONPs group was higher than that of the control, ZnONPs group (*P* < 0.001) and AMP group (*P* < 0.05). Therefore, AMP-ZnONPs could stimulate NO release more than AMP and ZnONPs in RAW264.7 cells, this suggested that ZnONPs displayed synergy with AMP.

#### AMP-ZnONPs induced cells cytokines secretion

Cytokines are the central logical targets for immune modulation as they influence the formation of a phenotype. They act as immunoregulators by either inducing or suppressing the production and maturation of immune cells (50). IL-1β is a major mediator of inflammation secreted by various activated innate immune cells, such as macrophages, monocytes and dendritic cells (51). IL-6 is also one of the important mediators that can stimulate antibody production and participate in immune response. Both IL-1β and IL-6 are of great importance for immune homeostasis and barrier immunity. To further investigate the immunological activity of AMP-ZnONPs on RAW264.7 cells, the cytokine (IL-1β and IL-6) contents in cells were evaluated by RT-qPCR in this study (Figures 6C,D). LPS stimulated the production of IL-1β and IL-6 by more than 2,000 times compared to the control group (*P* < 0.001), which indicated that LPS could promote inflammation and lead to excessive cytokine release (52). Notably, AMP-ZnONPs treatment exerted a significant action on IL-1β and IL-6 secretion than both AMP and ZnONPs (*P* < 0.001), which showed ZnONPs exerted a synergistic effect with AMP. The results indicated that the AMP-ZnONPs had immunostimulatory effect, but it did not cause cell inflammation like LPS.

#### AMP-ZnONPs enhanced cells phagocytosis

**FIGURE 7:**
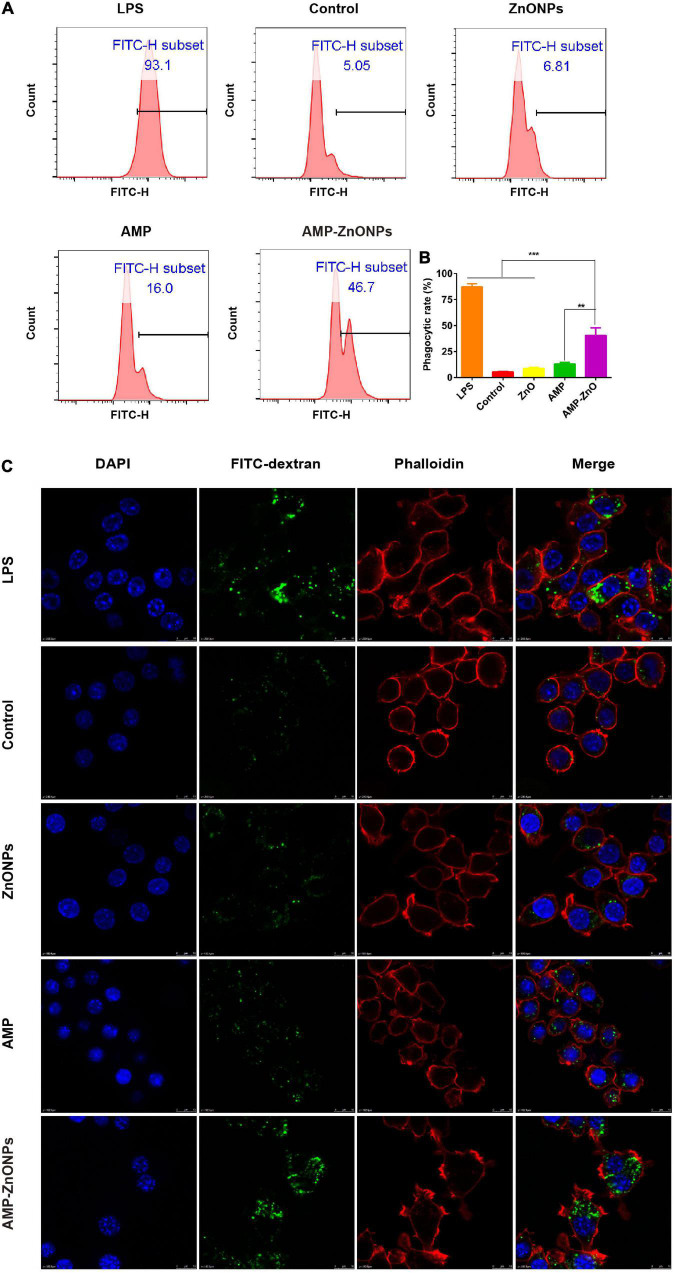
The phagocytosis was evaluated by flow cytometry **(A)**. The histogram showed the positive rate of cells for FITC-dextran **(B)**. (**P < 0.01, ***P < 0.001 vs. AMP-ZnONPs). Enhanced FITC-dextran uptake into RAW264.7 cells following incubation with AMP-ZnONPs. FITC-dextran (green) was mixed with ZnONPs, AMP, AMP-ZnONPs, LPS and DMEM medium (control) overnight avoiding light. Cytoskeleton and cell nuclei were stained with phalloidin (red) and DAPI (blue) respectively, **(C)**.

Macrophages exist in virtually all tissues, phagocytosis, which is a classic index to evaluate macrophage activation, plays a critical role in the uptake and degradation (53–55). In addition, it is a signal-inducing process in which phagosomes bind to the antibody on the cell surface, consequently, cell morphology and signaling pathways are affected (56). The enhanced phagocytosis is one of the remarkable characteristics of activated macrophages, meanwhile indicating the activation of innate immunity. The results showed that compared with the control and ZnONPs, AMP-ZnONPs and AMP could significantly promote cell phagocytosis of macrophages (Figures 7A,B). Meanwhile, it should be highlighted that the stimulating effect of AMP-ZnONPs on phagocytosis was remarkably higher than AMP (*P* < 0.05). Furthermore, FITC-dextran accumulation in RAW264.7 was measured by LSCM. As shown in Figure 7C, AMP-ZnONPs treatment markedly enhanced the fluorescence intensity of tested cells relative to AMP and ZnONPs, and the FITC-dextran were mainly distributed in the cytoplasm. These results demonstrated that the AMP binding with ZnONPs significantly improved the immune activity of RAW264.7, which indicated ZnONPs and AMP acted in synergy of immune system.

#### AMP-ZnONPs promoted cells costimulatory molecules expression

**FIGURE 8:**
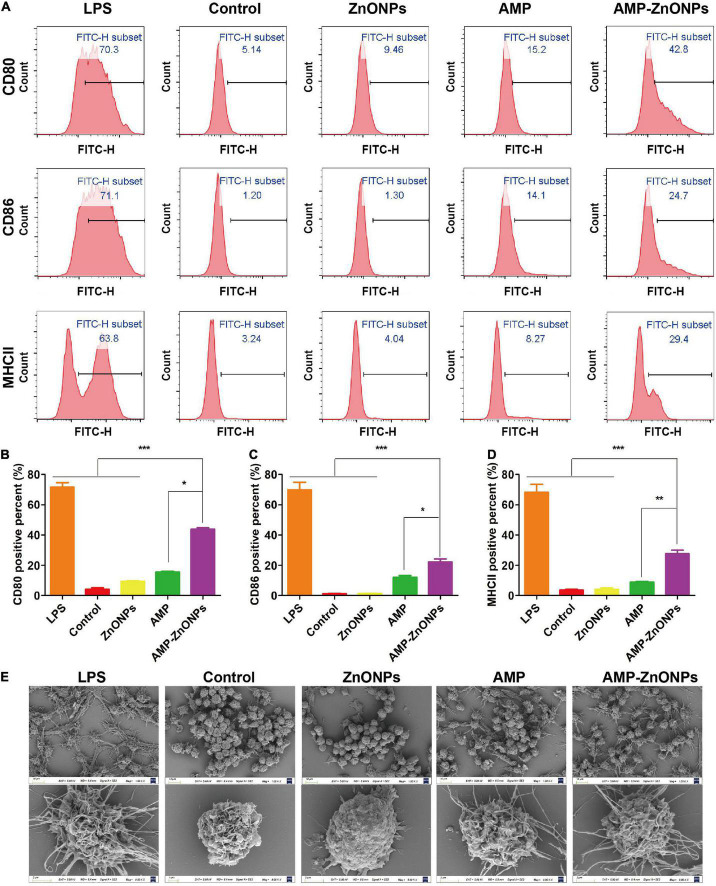
The production of CD80, CD86, MHCII in RAW264.7 cells were analyzed by flow cytometry **(A)**. The histogram showed the positive rate of CD80 **(B)**, CD86 **(C)**, MHCII **(D)** in cells. (*P < 0.05, **P < 0.01, ***P < 0.001 vs. AMP-ZnONPs). SEM analysis for morphological changes of RAW264.7 cells in different groups **(E)**.

The activation and further differentiation of T cells and cellular immune function are closely related to the function of antigen-presenting cells (APC), especially macrophages, considered as professional antigen-presenting cells (57). CD80 and CD86 may differentially control the T-cell activation because of the distinct properties of each molecule. Once presented to T cells by MHCII, peptide antigens generally stimulate a typical T cell-dependent immune response and the induction of immune memory (58). In this study, the expression of phenotypic markers of CD80, CD86 and MHCII up-regulated after the RAW264.7 cells were exposed to LPS for 24 h (Figure 8A). Figures 8B–D documented a significant increase in the percent of RAW264.7 cells positive for the expression of CD80, CD86, and MHCII following incubation with AMP-ZnONPs as compared to the control, ZnONPs and AMP. The results proved that AMP-ZnONPs significantly increased the expression of CD80, CD86 and MHCII compared with ZnONPs or AMP alone, which indicated ZnONPs and AMP showed synergetic effect with each other.

#### AMP-ZnONPs induced cells morphological changes

Macrophages engulf nutrients and pathogens by stretching their arms (59). The morphology was observed by SEM as shown in Figure 8E. Cells in the control group had a round shape and microvilli-like structures on the cell surface. RAW264.7 cells showed elongated and polygonal with many filopodia in the LPS group than in the other groups. In the AMP-ZnONPs group, even in the case of round-shaped RAW264.7 cells, their arms were stretching in various directions. The size, microvilli-like structures and surface folds of the AMP-ZnONPs treated group were more than ZnONPs and AMP. These results indicated that AMP-ZnONPs could induce RAW264.7 cells activation visibly, which was inconsistent with the results of phagocytosis and cytokine secretion.

#### AMP-ZnONPs regulated the expression of TLR4/MyD88/NF-κB associated proteins

**FIGURE 9:**
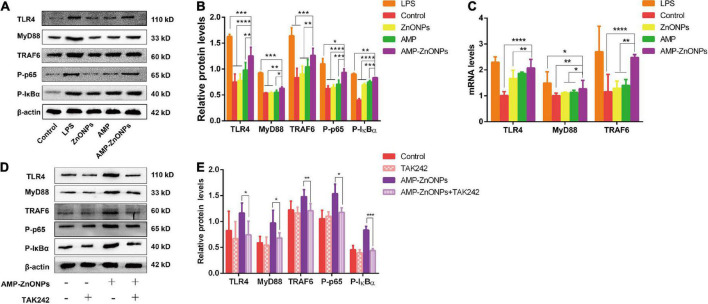
The relative expressions of proteins **(A,B)** and mRNAs **(C)** of the critical nodes in the TLR4/MyD88/NF-κB signaling pathways. Effects of TAK242 on TLR4, MyD88, TRAF6, P-IκBα and P-p65 expression stimulated by AMP-ZnONPs or not **(D,E)**. The results were expressed as the mean ± SD (*n* = 3) (*P < 0.05, **P < 0.01, ***P < 0.001, ****P < 0.0001 vs. AMP-ZnONPs).

As an important member of the TLR family, TLR4 has been widely reported to recognize and bind to different pathogen-related molecular patterns, initiate intracellular signal transduction pathways, cause the release of cytokines or chemokines, and play an effective innate immune response (60). Whether AMP-ZnONPs could mediate the immunomodulatory effect on RAW264.7 cells by the TLR4 signaling pathway was explored. The RT-qPCR and Western blot were used to detect the mRNA and proteins of key nodes in the TLR4/MyD88/NF-κB signaling pathways. As shown in Figures 9A,B, compared with AMP group, the protein expression levels of TLR4, MyD88, TRAF6, P-IκBα and P-p65 were upregulated in AMP-ZnONPs group. As shown in Figure 9C, AMP-ZnONPs significantly up-regulated the mRNA expression of TLR4, MyD88, TRAF6 in the RAW264.7 cells, compared with those in control, ZnONPs and AMP group. Therefore, the results indicated that AMP-ZnONPs was more effective than ZnONPs and AMP in activating the TLR4/MyD88/NF-κB signaling pathway.

#### AMP-ZnONPs downregulated expression of TLR4/MyD88/NF-κB associated proteins after treating with the TLR4 blocker TAK242

TAK242 is a specific inhibitor of TLR4 that affects the downstream signal transduction of TLR4 by interfering with the intracellular segment of TLR4 (61). In order to further verify that AMP-ZnONPs could activate the TLR4/MyD88/NF-κB signaling pathway, RAW264.7 cells were treated with the TLR4 antagonist TAK242, and the expression of key proteins in the pathway were detected. After adding TAK242, the expression of each protein in the TLR4 pathway was shown in Figure 9D. Compared with the control group, the expression of TLR4, MyD88, TRAF6, P-IκBα and P-p65 were increased after treatment with AMP-ZnONPs (Figure 9B), while the expression of the above proteins was significantly reduced after TAK242 was added (Figure 9E).

**FIGURE 10:**
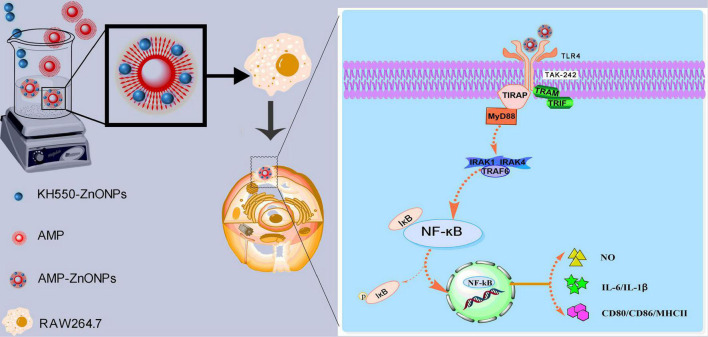
Schematic illustration depicts that a new approach was carried out to bind AMP with ZnONPs as an immunostimulator. AMP-ZnONPs could significantly activate RAW264.7 cells by TLR4/MyD88/NF-κB signaling pathway to improve its immune function. Therefore, AMP-ZnONPs remarkably enhanced phagocytosis, the release of NO, cytokines and the costimulatory molecules of RAW264.7 cells.

TLR4 generally signals *via* a MyD88-dependent pathway, then IKK phosphorylates IκB and p65 resulting in degradation of IκB and activation of NF-κB, a nuclear factor that is responsible for the production of many cytokines (IL-6, IL-1β) and the costimulatory molecules (CD80, CD86, MHCII). In this study, AMP-ZnONPs could significantly activate RAW264.7 cells by TLR4/MyD88/NF-κB signaling pathway to improve its immune function. TAK242, a specific TLR4 inhibitor, reversely demonstrated that AMP-ZnONPs could promote the expression of TLR4 pathway-related proteins. In conclusion, this study can provide a new research idea for the development and utilization of polysaccharides and microelements in the food and pharmaceutical industry and presents a theoretical basis for research and development into new immunomodulatory nutraceutical or immune adjuvant (Figure 10).

## Conclusion

In summary, by Borch reaction between AMP and ZnONPs modified by KH550, the AMP-ZnONPs was successfully prepared and its characterization was evaluated. AMP-ZnONPs showed excellent immunostimulatory activity on macrophages and the activities were much better than those of ZnONPs or AMP applied alone. Furthermore, this study clarified that AMP-ZnONPs could significantly activate RAW264.7 cells by TLR4/MyD88/NF-κB signaling pathway to improve its immune function. These data demonstrated that AMP binding with ZnONPs could potentially be used as an easily available source for immunomodulatory nutraceutical or immune adjuvant, which can be widely used in the food or medicine industry in the future.

## Data availability statement

The datasets presented in this study can be found in online repositories. The names of the repository/repositories and accession number(s) can be found in the article/supplementary material.

## Author contributions

RB: conceptualization, methodology, software, data curation, writing—original draft, review and editing, visualization, supervision, project administration, and funding acquisition. XL: conceptualization, methodology, investigation, software, data curation, writing—original draft, and visualization. JW: methodology, investigation, and software. SW: investigation and writing—review and editing. XW: investigation and software. YT and SX: visualization and investigation. ML: supervision. JL: conceptualization, methodology, project administration, and funding acquisition. HP: conceptualization and methodology. All authors contributed to the article and approved the submitted version.
